# Supplementary material for: RudLOV is an optically synchronized cargo transport method revealing unexpected effects of dynasore
Source: EMBO Rep. 2024 Dec 10;26(3):613–34. doi: 10.1038/s44319-024-00342-z (PMC11811055; doi:10.1038/s44319-024-00342-z)
Supplement: Supplementary file 1 — Appendix [file 44319_2024_342_MOESM1_ESM.pdf]

APPENDIX

Table of content

Content	*Page number*
Table of content	1
Appendix Figure S1	2
Appendix Figure S2	3

Tago et al., Appendix Figure S1

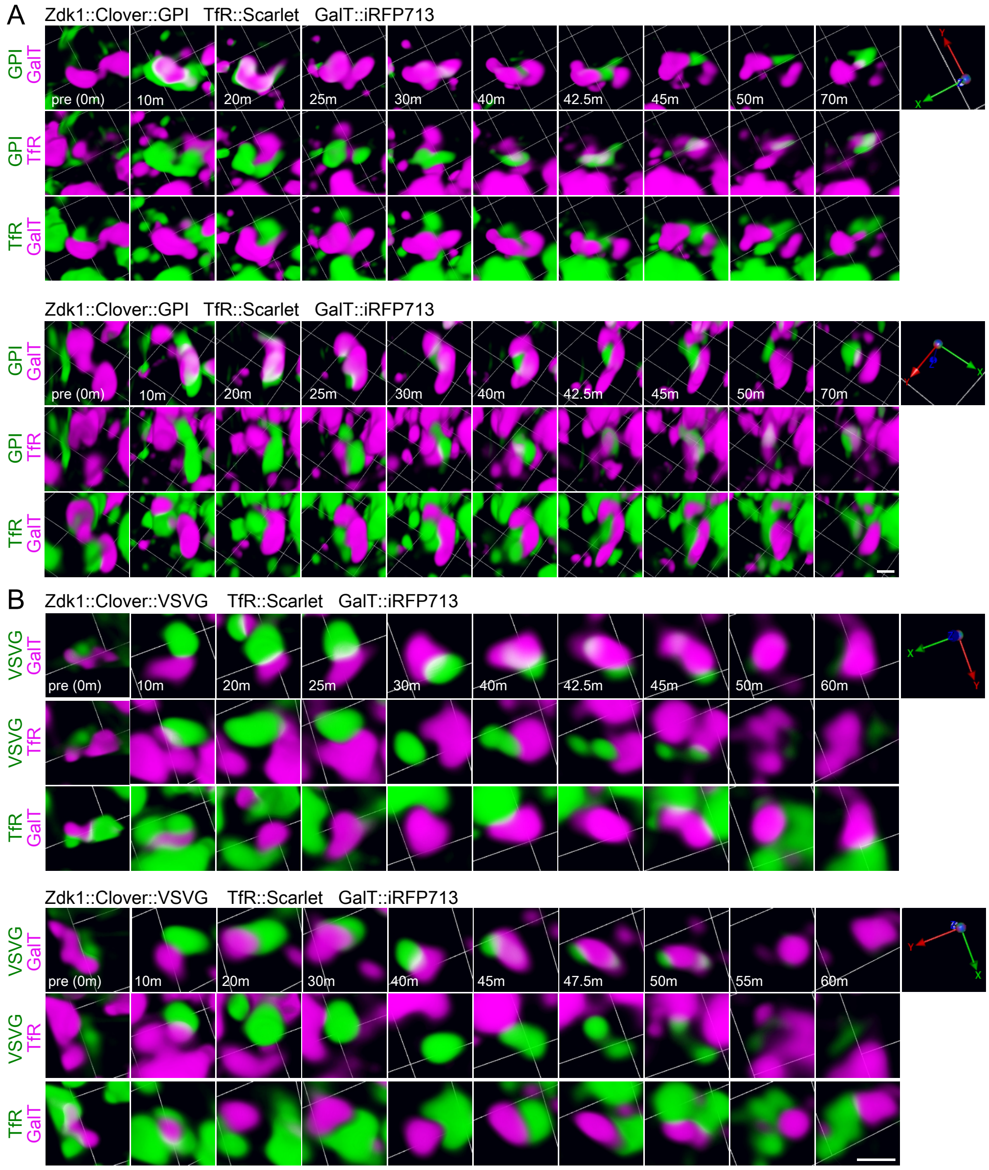

Appendix Figure S1. GPI–AP and VSVG movements within Golgi/RE units observed by RudLOV

(A) Double-colored images separated from triple-colored images in Figure 2E.

(B) Double-colored images separated from triple-colored images in Figure 2I.

Data in A and B are representative of more than three replicates.

Scale bars: 1 µm (A, B).

Tago et al., Appendix Figure S2

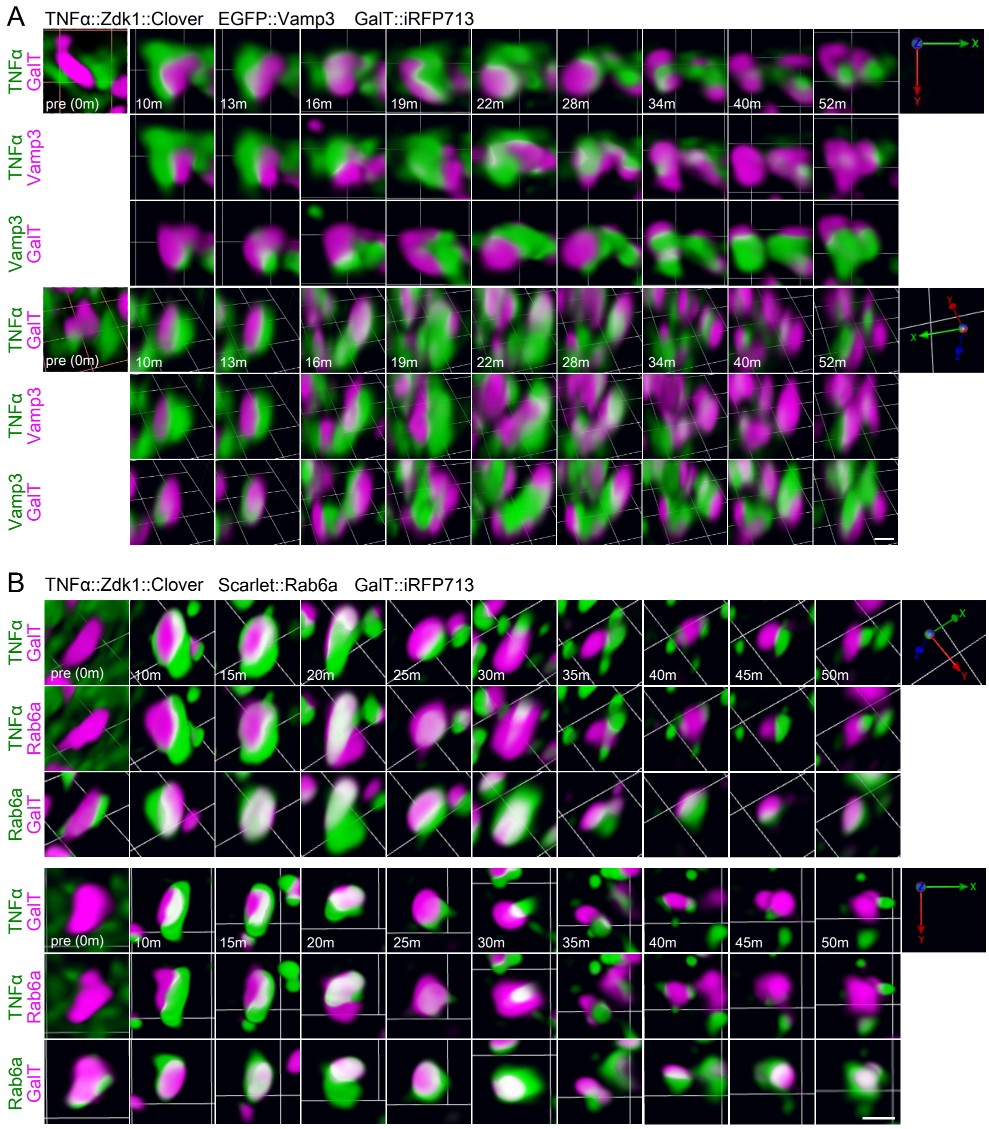

Appendix Figure S2. TGNα movements within Golgi/RE unit observed by RudLOV

(A) Double-colored images separated from triple-colored images in Figure 2M.

(B) Double-colored images separated from triple-colored images in Figure 2Q

Data in A and B are representative of more than three replicates.

Scale bars: 1 μm (A, B).
