# Supplementary material for: RudLOV is an optically synchronized cargo transport method revealing unexpected effects of dynasore
Source: EMBO Rep. 2024 Dec 10;26(3):613–34. doi: 10.1038/s44319-024-00342-z (PMC11811055; doi:10.1038/s44319-024-00342-z)
Supplement: Supplementary file 5 — Expanded View Figures [file 44319_2024_342_MOESM5_ESM.pdf]

Expanded View Figures

Figure EV1. Delay of cargo movement in cells with high hook/cargo expression in the RUSH system.

(**A**) Double-colored images with SBP::NeonGreen::VSVG (green) and GalT::iRFP713 (magenta) of Fig. 1A. (**B**) Dot plot of the relative expression level of the cargo in each cell, categorized by different time-to peak of SBP::NeonGreen::VSVG after biotin administration in the Golgi apparatus in the RUSH system. (**C**) Localization of SBP::NeonGreen::VSVG against Golgi stack at 0 min (left) and 10 min (right) after the first observation (0 min) without transport-trigger. Golgi markers (TagBFP2::GM130, GalT::iRFP713) and ERES marker (Scarlet::Sec13) are shown in the indicated colors. Plots show signal intensities from the image on the upper ERES/Golgi unit. Signal intensity was measured along the arrow (representing 1.5 µm). (**D**) Double-colored images with Zdk1::Clover::VSVG (green) and GalT::iRFP713 (magenta) of Fig. 1F. (**E**, **F**) Localization of Zdk1::Clover::GPI before (left) and at 10, 15, 30, 40, 50, and 70 min after onset of illumination at 445 nm using the RudLOV system in untreated (**E**) and nocodazole-treated cells (**F**). Inset in (**F**) shows a magnified image of Zdk1::Clover::GPI. The cargo is shown in green and GalT::iRFP713 in magenta. (**G**) Localizations of TNFα::Zdk1::Clover before (left) and at 10, 15, 20, 30, 50, and 60 min after onset of illumination with 445 nm using the RudLOV system. The cargo is shown in green, Ruby::GM130 in red, and GalT::iRFP713 in blue. (**H**) Localizations of TNFα::Zdk1::Clover before (left) and at 10, 20, 30, 50, and 70 min after onset of illumination with 445 nm using the RudLOV system in nocodazole-treated cells. Inset shows the magnified image of TNFα::Zdk1::Clover. The cargo is shown in green, Ruby::GM130 in red, and GalT::iRFP713 in blue. Data in (**A**) and (**C**–**H**) are representative of more than three replicates. Scale bars: 20 µm (**A**, **D**), 5 µm (**E**–**H**), and 2 µm (insets in **F**, **H**).

© The Author(s)

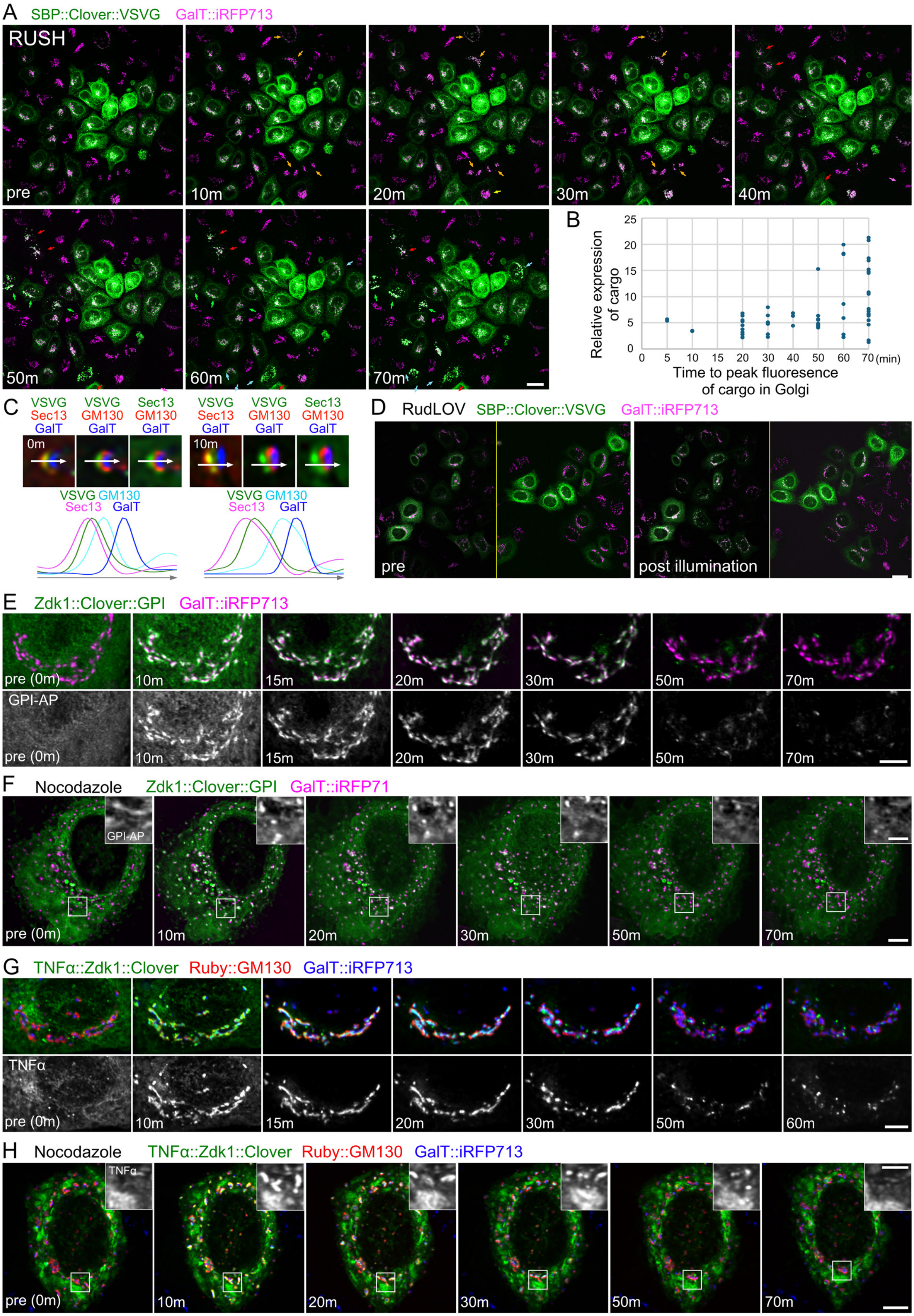

A SBP::Clover::VSVG GalT::iRFP713
RUSH
pre
10m
20m
30m
40m
50m
60m
70m
B
Relative expression of cargo
Time to peak fluoresence of cargo in Golgi
(min)
C
VSVG Sec13 GalT
VSVG GM130 GalT
Sec13 GM130 GalT
0m
10m
VSVG GM130 Sec13 GalT
D RudLOV SBP::Clover::VSVG GalT::iRFP713
pre
post illumination
E Zdk1::Clover::GPI GalT::iRFP713
pre (0m)
10m
15m
20m
30m
50m
70m
GPI-AP
F Nocodazole Zdk1::Clover::GPI GalT::iRFP71
GPI-AP
pre (0m)
10m
20m
30m
50m
70m
G TNFα::Zdk1::Clover Ruby::GM130 GalT::iRFP713
TNFα
pre (0m)
10m
15m
20m
30m
50m
60m
H Nocodazole TNFα::Zdk1::Clover Ruby::GM130 GalT::iRFP713
TNFα
pre (0m)
10m
20m
30m
50m
70m

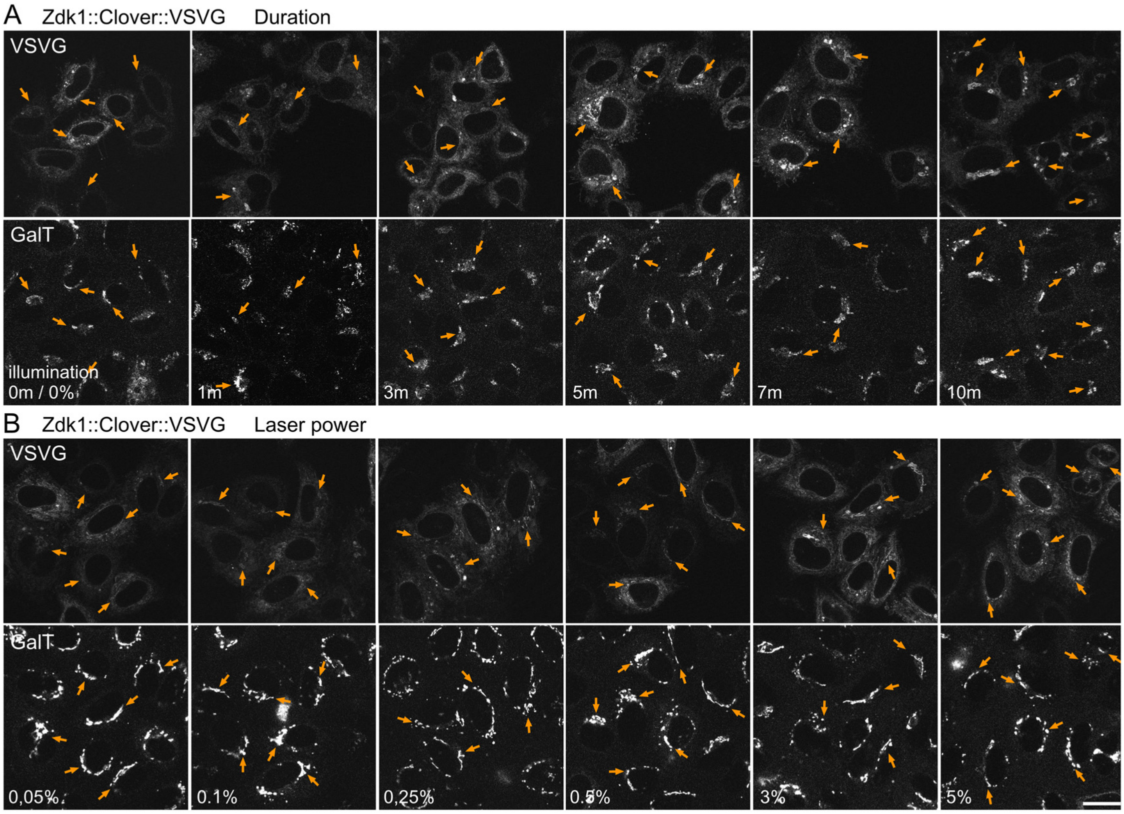

Figure EV2. RudLOV enables quantitative control of cargo release.

(**A**) Localization of Zdk1::Clover::VSVG (upper panel) or GalT::iRFP713 (lower panel) 10 min after the start of 0, 1, 3, 5, 7, and 10 min illumination at 445 nm with 3% laser power using the RudLOV system. Arrows indicate Golgi stacks. (**B**) Localization of Zdk1::Clover::VSVG (upper panel) or GalT::iRFP713 (lower panel) after 5 min illumination at 445 nm with 0.05%, 0.1%, 0.25%, 0.5%, 3%, and 5% laser power using the RudLOV system. Arrows indicate Golgi stacks. Data in (**A**) and (**B**) are representative of more than three replicates. Scale bars: 20 µm (**A**, **B**).

© The Author(s)

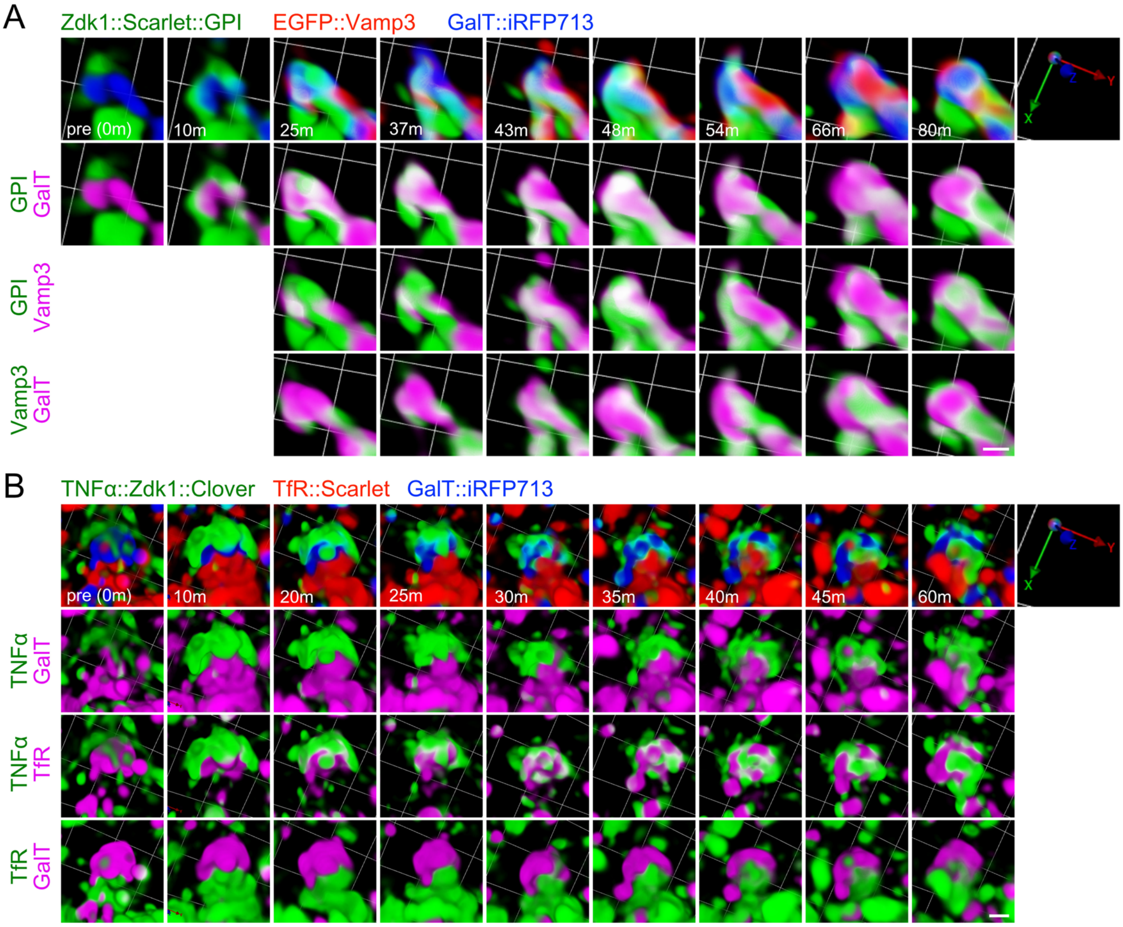

Figure EV3. TNFα movements within Golgi/RE unit observed by RudLOV.

(**A**) Localization of the cargo Zdk1::Scarlet::GPI before (left) and after illumination in a single Golgi/RE unit in nocodazole-treated cells. The time after illumination is shown in the bottom-left corner. The cargo is shown in green, *trans*-Golgi marker GalT::iRFP713 in blue, and RE marker EGFP::Vamp3 in red (upper panel). Double-colored images separated from triple-colored images (lower panels). (**B**) Localization of the cargo TNFα::Zdk1::Clover before (left) and after illumination in a single Golgi/RE unit in nocodazole-treated cells. The time after illumination is shown in the bottom-left corner. The cargo is shown in green, *trans*-Golgi marker GalT::iRFP713 in blue, and RE marker TfR::Scarlet in red (upper panel). Double-colored images separated from triple-colored images (lower panels). Data in (**A**) and (**B**) are representative of more than three replicates. Scale bars: 1 µm (**A**, **B**).

 © The Author(s)

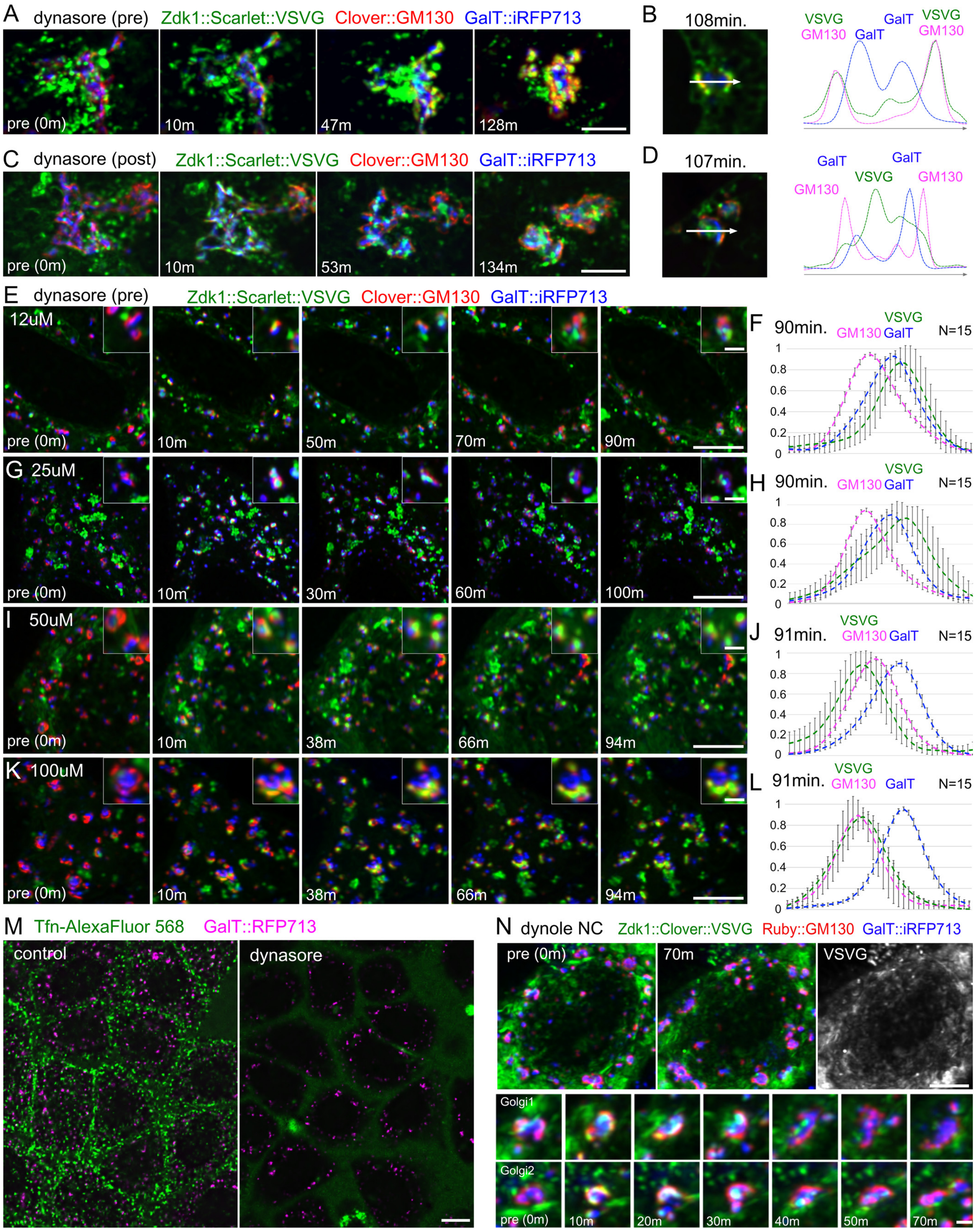
A dynasore (pre) Zdk1::Scarlet::VSVG Clover::GM130 GalT::iRFP713
pre (0m)
10m
47m
128m
B 108min.
VSVG
GM130
GalT
GalT
VSVG
GM130
C dynasore (post) Zdk1::Scarlet::VSVG Clover::GM130 GalT::iRFP713
pre (0m)
10m
53m
134m
D 107min.
GalT
GalT
GM130
VSVG
GM130
E dynasore (pre) Zdk1::Scarlet::VSVG Clover::GM130 GalT::iRFP713
12uM
pre (0m)
10m
50m
70m
90m
F 90min.
VSVG
GM130 GalT
N=15
G 25uM
pre (0m)
10m
30m
60m
100m
H 90min.
VSVG
GM130 GalT
N=15
I 50uM
pre (0m)
10m
38m
66m
94m
J 91min.
VSVG
GM130 GalT
N=15
K 100uM
pre (0m)
10m
38m
66m
94m
L 91min.
VSVG
GM130 GalT
N=15
M Tfn-AlexaFluor 568 GalT::RFP713
control
dynasore
N dynole NC Zdk1::Clover::VSVG Ruby::GM130 GalT::iRFP713
pre (0m)
70m
VSVG
Golgi1
Golgi2
pre (0m)
10m
20m
30m
40m
50m
70m

Figure EV4. Dynasore inhibits cargo transport at the *cis*- or *trans*-side of Golgi stacks in nocodazole-untreated cells.

(**A**) Localization of Zdk1::Scarlet::VSVG before illumination and at 10, 47, and 128 min after onset of illumination in cells pre-treated with dynasore. The cargo is in green, *cis*-Golgi marker Clover::GM130 in red, and *trans*-Golgi marker GalT::iRFP713 in blue. (**B**) Plots showing signal intensities from the image of the left Golgi/RE unit as 108 min after onset of illumination in the cell pre-administered with dynasore. Signal intensity was measured along the arrow (representing 3 μm). (**C**) Localization of Zdk1::Scarlet::VSVG before illumination and at 10, 53, and 134 min after onset of illumination in cells that were post-administrated dynasore 4 min after illumination. The cargo is in green, *cis*-Golgi marker Clover::GM130 in red, and *trans*-Golgi marker GalT::iRFP713 in blue. (**D**) Signal intensities from the image of the left Golgi/RE unit 107 min after onset of illumination in a dynasore pre-administered cell. Signal intensity was measured along the arrow (representing 3 μm). (**E–L**) Localization of Zdk1::Scarlet::VSVG before illumination (left) and after illumination in cells pre-treated with dynasore at 100, 50, 25, and 12 μM (**E**, **G**, **I**, **K**). Insets in (**E**), (**G**), (**I**), and (**K**) show the magnified image of a single Golgi stack. Dynasore was administrated 1 min before illumination. Zdk1::Scarlet::VSVG is shown in green, *cis*-Golgi marker Clover::GM130 in red, and *trans*-Golgi marker GalT::iRFP713 in blue. Plots show the normalized means of 15 line profiles of Zdk1::Scarlet::VSVG, Clover::GM130 and GalT::iRFP713 across the Golgi stack at 90 min (**F**, **H**) and 91 min (**J**, **L**) after the start of illumination. The 15 line profiles were obtained from five Golgi stacks per cell, using three different cells. Error bars are presented as mean ± SD. (**M**) Uptake of Tfn in untreated and dynasore-treated cells 8 min after incubation with 30 μg/ml of Alexa Fluor 568-conjugated Tfn. (**N**) Localization of Zdk1::Clover::VSVG before illumination (upper left) and 70 min after illumination (upper middle and right) in cells pre-treated with dynole 31-2 (negative control: NC). Time-course of Zdk1::Clover::VSVG localization before and after illumination in a dynole NC-treated single Golgi stack (bottom). Zdk1::Clover::VSVG is shown in green, *cis*-Golgi marker Ruby::GM130 in red, and *trans*-Golgi marker GalT::iRFP713 in blue. Data in (**A**), (**C**), (**E**), (**G**), (**I**), (**K**), (**M**) and (**N**) are representative of more than three replicates. Scale bars: 5 μm (**A**, **C**, **E**, **G**, **I**, **K**), 1 μm (insets in **E**, **G**, **I**, **K**), 10 μm (**M**), 5 μm (upper panel in **N**), and 1 μm (lower panel in **N**).

© The Author(s)

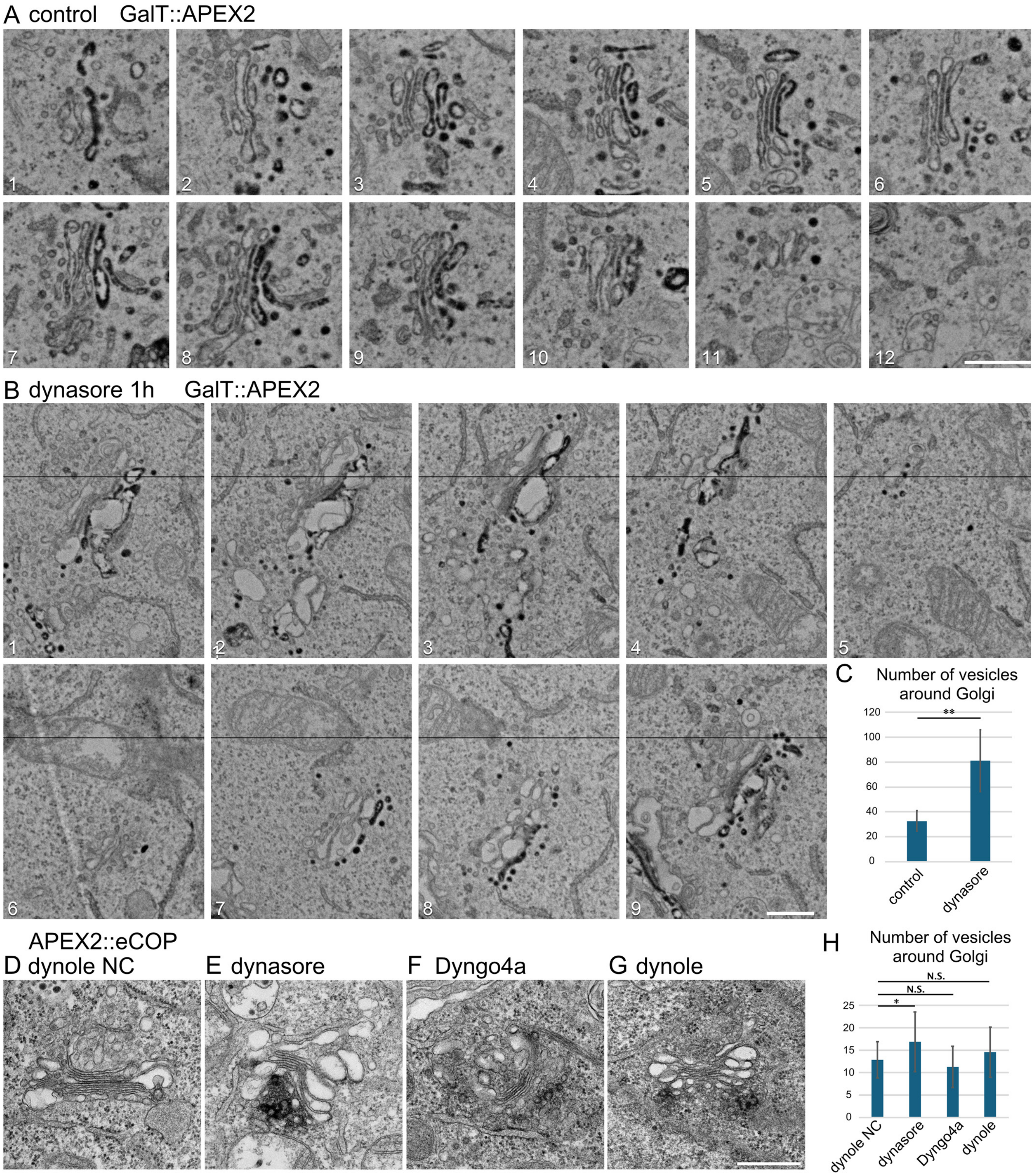
A control GalT::APEX2
1
2
3
4
5
6
7
8
9
10
11
12
B dynasore 1h GalT::APEX2
1
2
3
4
5
6
7
8
9
C
Number of vesicles around Golgi
**
120
100
80
60
40
20
0
control
dynasore
APEX2::eCOP
D dynole NC
E dynasore
F Dyngo4a
G dynole
H
Number of vesicles around Golgi
N.S.
N.S.
*
25
20
15
10
5
0
dynole NC
dynasore
Dyngo4a
dynole

Figure EV5. Accumulation of vesicles at the *cis*-side of Golgi stacks after administration of dynasore.

(**A**, **B**) Scanning electron micrographs of serial sections of a Golgi stack at 150 nm-intervals in the cell after 1 h of incubation with (**B**) or without 100 μM dynasore (**A**). GalT::APEX2 visualized trans-Golgi cisternae and vesicles. (**C**) Number of vesicles accumulated near Golgi stacks in the cell after 1 h of incubation with or without 100 μM dynasore. Error bars indicate the standard deviation of five Golgi stacks. Significance according to two-tailed unpaired Student's t-test (Welch's t-test), where $^{**}p < 0.01$. Error bars are presented as mean ± SD. Exact *P* values (to 4 decimal points) for (**C**) 0.0072. (**D–G**) Transmission electron micrographs of Golgi stacks with APEX2::eCOP showing COPI budding profiles and vesicles in the cell after 1 h of incubation with dynole NC (**D**), dynasore (**E**), Dyngo4a (**F**), and dynole (**G**). (**H**) Number of vesicles accumulated near Golgi stacks in the cross sections of cells after 1 h of incubation with dynasore, dynole NC, dynole, or Dyngo4a. Error bars indicate the standard deviation of $N > 13$ sections of Golgi stacks. Significance according to two-tailed unpaired Student's t-test (Welch's t-test), where $^{*}p < 0.05$. Error bars are presented as mean ± SD. Exact *P* values (to 4 decimal points) for (**H**) dynoleNC vs dynasore 0.0444, dynoleNC vs Dyngo4a 0.3589 and dynoleNC vs dynole 0.3452. Data in (**A**), (**B**) and (**D–G**) are representative of more than three replicates. Scale bars: 500 nm (**A**, **B**, **D–G**).

 © The Author(s)
